# miR-127 Protects Proximal Tubule Cells against Ischemia/Reperfusion: Identification of Kinesin Family Member 3B as miR-127 Target

**DOI:** 10.1371/journal.pone.0044305

**Published:** 2012-09-04

**Authors:** Elia Aguado-Fraile, Edurne Ramos, David Sáenz-Morales, Elisa Conde, Ignacio Blanco-Sánchez, Konstantinos Stamatakis, Luis del Peso, Edwin Cuppen, Bernhard Brüne, María Laura García Bermejo

**Affiliations:** 1 Department of Pathology, Instituto Ramón y Cajal de Investigación Sanitaria (IRYCIS), Madrid, Spain; 2 Department of Cell Biology and Immunology, Centro de Biología Molecular Severo Ochoa (CBM-SO) (CSIC-UAM), Madrid, Spain; 3 Department of Biochemistry/HIV Unit, Hospital La Paz (IdiPAZ), Madrid, Spain; 4 Institute of Biomedical Research Alberto Sols, CSIC-UAM, Madrid, Spain; 5 Genome Biology Group, Hubrecht Institute, Utrecht, The Netherlands; 6 Pathobiochemistry, Faculty of Medicine, Goethe-University Frankfurt, Frankfurt, Germany; 7 Physiology Department, Alcalá University, Madrid, Spain; The Chinese University of Hong Kong, Hong Kong

## Abstract

Ischemia/reperfusion (I/R) is at the basis of renal transplantation and acute kidney injury. Molecular mechanisms underlying proximal tubule response to I/R will allow the identification of new therapeutic targets for both clinical settings. microRNAs have emerged as crucial and tight regulators of the cellular response to insults including hypoxia. Here, we have identified several miRNAs involved in the response of the proximal tubule cell to I/R. Microarrays and RT-PCR analysis of proximal tubule cells submitted to I/R mimicking conditions *in vitro* demonstrated that miR-127 is induced during ischemia and also during reperfusion. miR-127 is also modulated in a rat model of renal I/R. Interference approaches demonstrated that ischemic induction of miR-127 is mediated by Hypoxia Inducible Factor-1alpha (HIF-1α) stabilization. Moreover, miR-127 is involved in cell-matrix and cell-cell adhesion maintenance, since overexpression of miR-127 maintains focal adhesion complex assembly and the integrity of tight junctions. miR-127 also regulates intracellular trafficking since miR-127 interference promotes dextran-FITC uptake. In fact, we have identified the Kinesin Family Member 3B (KIF3B), involved in cell trafficking, as a target of miR-127 in rat proximal tubule cells. In summary, we have described a novel role of miR-127 in cell adhesion and its regulation by HIF-1α. We also identified for the first time KIF3B as a miR-127 target. Both, miR-127 and KIF3B appear as key mediators of proximal epithelial tubule cell response to I/R with potential al application in renal ischemic damage management.

## Introduction

microRNAs (miRNAs) are small (∼21 nucleotide-long), endogenous RNA molecules that have emerged as key post-transcriptional regulators of gene expression [1]. They are involved in a wide range of biological processes, including development, cell proliferation and differentiation, apoptosis and metabolism [1]–[3]. Bioinformatics approaches have described that, in mammals, they could regulate almost ∼50% of the protein-coding genes [1] and changes in their expression have been related to the pathogenesis of several human diseases [3].

In animals, most miRNAs are processed from longer hairpin transcripts by the action of two members of the RNAse III family of enzymes called Drosha and Dicer. This cleavage generates a ∼20 nucleotide miRNA/miRNA* duplex. One strand of the hairpin duplex is loaded into an Argonaute Family Protein (AGO) to form the miRNA-Induced silencing complexes (miRISCs) [1], [3]. As a part of these complexes, miRNAs silence the expression of target genes by translational repression or mRNA deadenylation and degradation [1]. Due to their ability to recognize hundreds of target mRNA and their reversible regulation, miRNAs have emerged as key controllers of rapid cell responses to environmental changes and stress [1], [4].

Ischemia/Reperfusion is one of the principal causes of Acute Tubular Necrosis, which underlies most of the cases of Acute Renal Failure. Sublethal ischemic injury is characterized by a rapid loss of proximal tubule cell polarity and cytoskeleton integrity. After ischemia, apical actin cytoskeleton is rapidly reorganized and adhesion molecules change their localization. These features lead to impairment of cell-cell and cell-matrix adhesion structures and cell detachment and consequently kidney dysfunction [5]–[8].

HIF-1α is a key modulator of cellular transcriptional response to low oxygen conditions and it activates a great number of metabolic and bioenergetic adaptative responses to hypoxic conditions [9].

HIF-1α plays an important role in kidney response to hypoxia [10]. It promotes changes in gene expression involved in angiogenesis and tissue repair after ischemic insult. Previous data of our laboratory demonstrated that *in vivo* inhibition of HIF-1α in a rat model of renal ischemia/reperfusion aggravates ischemic injury [11]. Moreover, HIF-1α accumulation in the kidney has a protective effect against ischemic damage [12].

Ischemia induces marked changes in microRNA expression and there is accumulating evidence that HIF-1α is responsible for regulating several miRNAs involved in cell responses to hypoxia, such as miR-210 or miR-373 [13]. Moreover, miRNAs are modulated in several acute ischemic pathologies including ischemic renal damage [14], [15]. In fact, conditional knock-out of Dicer in kidney promotes resistance to I/R injury [16].

Given the importance of miRNAs in gene expression regulation and their implication in renal ischemia reperfusion injury, we have studied the expression of microRNAs using an *in vitro* model of Hypoxia/Reoxygenation (Figure S1 A) in proximal tubule cells from rat and an *in vivo* model of renal ischemia/reperfusion in rat. Our data suggest that miR-127, controlled by HIF-1α, is induced in response to Hypoxia/Reoxygenation both *in vitro* and *in vivo.* This miRNA plays an important role in cytoskeleton protection, in cellular trafficking regulation and proximal tubule cell function recovery. Notably, a new target for miR-127 has been identified in this work: Kinesin Family Member 3B (KIF3B).

## Results

### miR-127 is Induced in Response to H/R and I/R

Firstly, we performed an initial screening analysis using microarrays to identify miRNAs that could be regulated in response to H/R. This experiment led to a set of miRNAs that modulated their expression not only during hypoxia, but also during reoxygenation in our *in vitro* model in NRK-52E cells (Table 1).

**Table 1:** Differentially expressed miRNAs in NRK-52E cells submitted to H/R.

Control vs Hypoxia		Hypoxia vs Reoxygenation	
miRNA	Fold change	miRNA	Fold change
**rno-miR-101a**	1	**rno-miR-101a**	2,63
**rno-miR-127**	1,16	**rno-miR-127**	2,46
rno-miR-129*	1	rno-miR-129*	2,13
**rno-miR-154**	1	**rno-miR-154**	8,03
**rno-miR-28**	1,27	**rno-miR-28**	1,90
**rno-miR-376b**	1,33	**rno-miR-376b**	6,70
**rno-miR-223**	1	**rno-miR-223**	−1,91

microRNA profiling was performed by hybridization array experiments. Comparisons between hypoxia vs control condition as well as hypoxia vs reoxygenation were done. Only miRNAs with statistical significance (P<0.05) in at least one analysis are shown.

**Figure 1:**
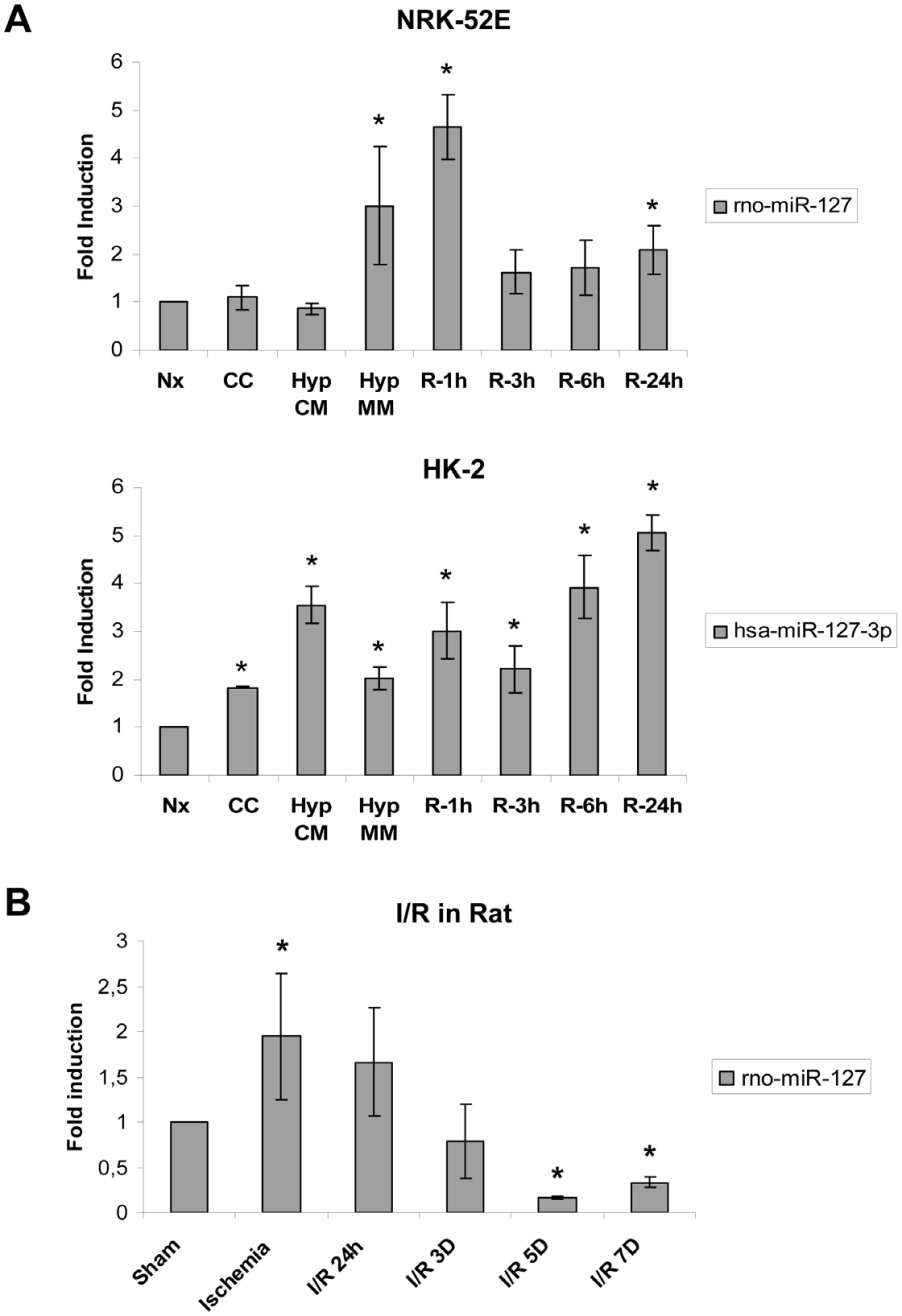
miR-127 is modulated in response to Hypoxia/Reoxygenation *in vitro* and during Ischemia/Reperfusión *in vivo*. (A) rno-miR-127 modulation in rat proximal tubule cells NRK-52E (Upper panel) and human proximal tubule cells HK-2 (Lower panel) submitted to hypoxia/reoxygenation (H/R) protocol. microRNA detection was performed by quantitative PCR using specific assays for miR-127 and RNU6B, which was employed as housekeeping control. Fold values were obtained by ΔΔCt method using Normoxia (Nx) as basal condition. Data are presented as mean±s.e.m. of five independent experiments in each panel. Asterisks indicate statistical significance comparing each sample with Normoxia (P<0.05). (B) rno-miR-127 modulation in rat kidney in response to ischemia/reperfusion (I/R). miRNA expression was estimated by quantitative PCR using specific taqman assays. Ribosomic RNA 5s was used as housekeeping gene and fold values were obtained comparing each group to Sham operated animals. Data are presented as mean±s.e.m. of at least five animals per condition. Asterisks indicate statistical significance (P<0.05) comparing each experimental group to Sham animals. (Nx: Normoxia; CC: Medium change control; Hyp CM: hypoxia in complete medium; Hyp MM: hypoxia in minimum medium; R-1h: Hypoxia in minimum medium and 1 Hour reoxygenation; R-3h: Hypoxia in minimum medium and 3 hours of reoxygenation; R-6h: Hypoxia in minimum medium and 6 hours of reoxygenation; R-24h Hypoxia in minimum medium and 24 hours of reoxygenation; I/R-24h: ischemia and 24 hours of reperfusion; I/R 3D: ischemia and 3 days of reperfusion; I/R 5D: ischemia and 5 days of reperfusion; I/R 7D: ischemia and 7 days of reperfusion).

Next, we validated these microarray data by qPCR. The rno-miR-127 was the most consistent and significantly modulated miRNA showing an increased expression during minimum medium hypoxia (mimicking ischemia) and 1 hour of reperfusion (Figure 1A). The human homolog of this miRNA (hsa-miR-127-3p) is also induced in HK-2 cells but showing a different expression pattern. In this case, we found increased expression during complete medium hypoxia and along reoxygenation. Moreover, rno-miR-127 is also induced during ischemia and 24 hours of reperfusion in our *in vivo* rat model of I/R (Figure 1B). Representative histology images for the in vivo model as well as creatinine and urea values, indicating renal injury, can be found in Figure S2. Proximal tubule cell detachment, distalization of proximal tubules and hyaline casts can be observed at I/R 24 h, when ischemic damage is maximal. These features correlate with a significant increase in serum creatinine and urea values. At I/R 7D kidney structure as well as function is recovered. This *in vivo* model has been widely used and characterized for renal I/R injury studies [11], [17], [18].

Taken together, these data indicate that miR-127 is modulated in proximal tubule cells and renal tissue in response to H/R and I/R.

### hsa-miR-127 is Regulated During H/R by HIF-1α

As HIF-1α is a key regulator of the cell response to hypoxia, we determined if this transcription factor could be involved in the modulation of miR-127 in our system.

**Figure 2:**
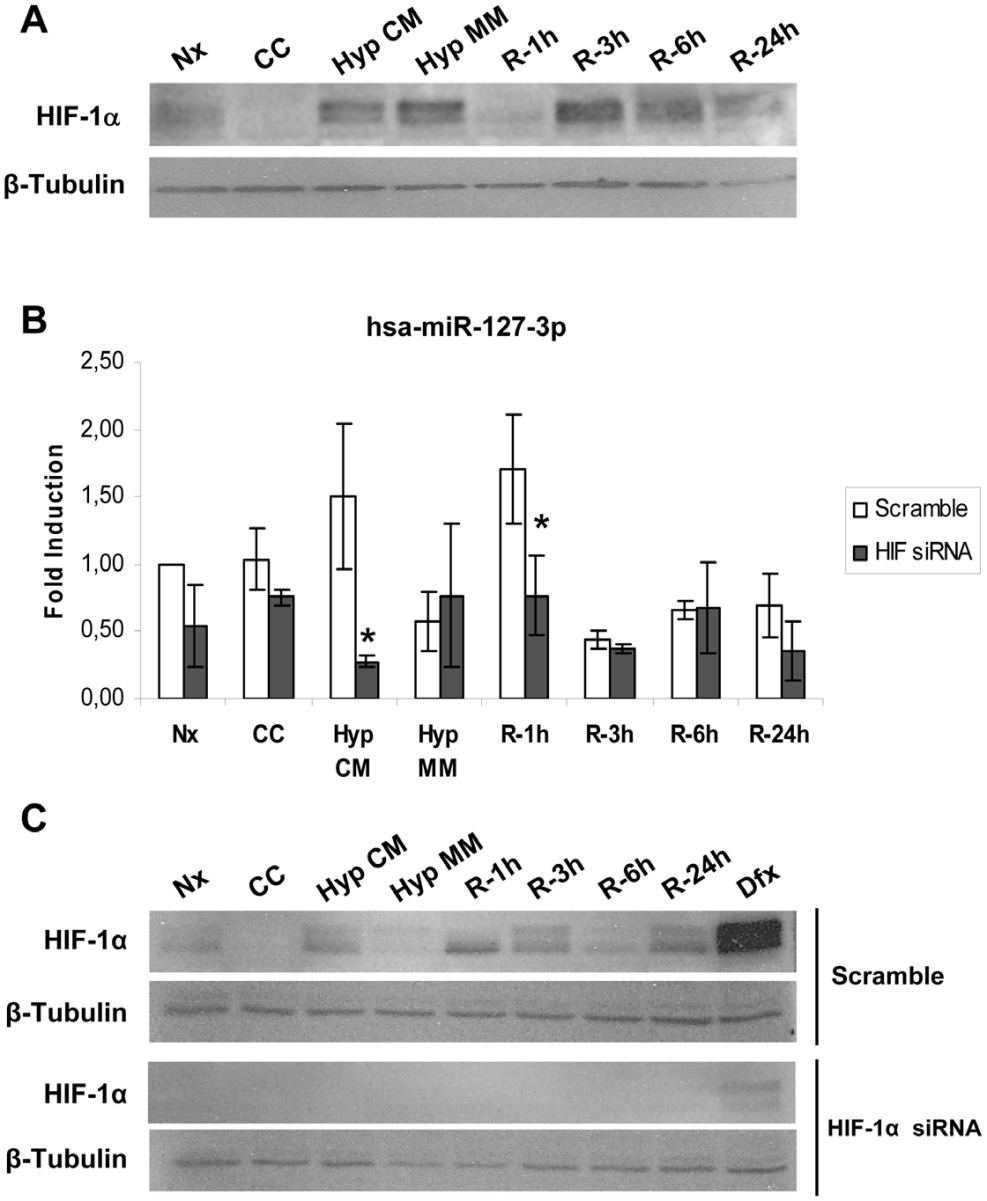
HIF-1α regulates miR-127-3p in HK-2 cells in response to Hypoxia/Reoxygenation. (A) HIF-1α stabilization in HK-2 cells during H/R protocol was estimated by western blot using β-tubulin as loading control (lower panel). Representative image from five independent experiments is shown. (B) hsa-miR-127-3p expression in HK-2 cells transfected with scramble (White bars) or HIF-1α siRNA (Black bars). microRNA expression was determined by quantitative PCR using specific assays. RNU6B was used as a housekeeping control and fold values were obtained comparing each sample to Normoxia scramble (Nx). Data are presented as mean±s.e.m. of five independent experiments. Asterisks indicate statistical significance (P<0.05) comparing scramble to siRNA values in each condition. (C) Interference efficiency was estimated by HIF-1α detection by western blot. Upper panel shows HIF-1α stabilization in scramble transfected cells submitted to H/R whereas lower panel indicates HIF-1α protein levels in siRNA transfected cells. β-tubulin was used as loading control. Deferoxamine was used as a positive control for HIF-1α stabilization. Representative western blot image from five experiments is shown. (Nx: Normoxia; CC: Medium change control; Hyp CM: hypoxia in complete medium; Hyp MM: hypoxia in minimum medium; R-1h: Hypoxia in minimum medium and 1 Hour reoxygenation; R-3h: Hypoxia in minimum medium and 3 hours of reoxygenation; R-6h: Hypoxia in minimum medium and 6 hours of reoxygenation; R-24h Hypoxia in minimum medium and 24 hours of reoxygenation; Dfx: deferoxamine).

In our *in vitro* model, HIF-1α is expressed not only during hypoxia, but also at several time points during reperfusion, showing a biphasic induction pattern as previously published [11] (Figure 2A). Knockdown of this factor by siRNA transfection successfully prevented miR-127-3p induction during complete medium hypoxia and 1 hour of reperfusion in HK-2 cells (Figure 2B). HIF-1α interference control western blot is shown in figure 2C.

**Figure 3:**
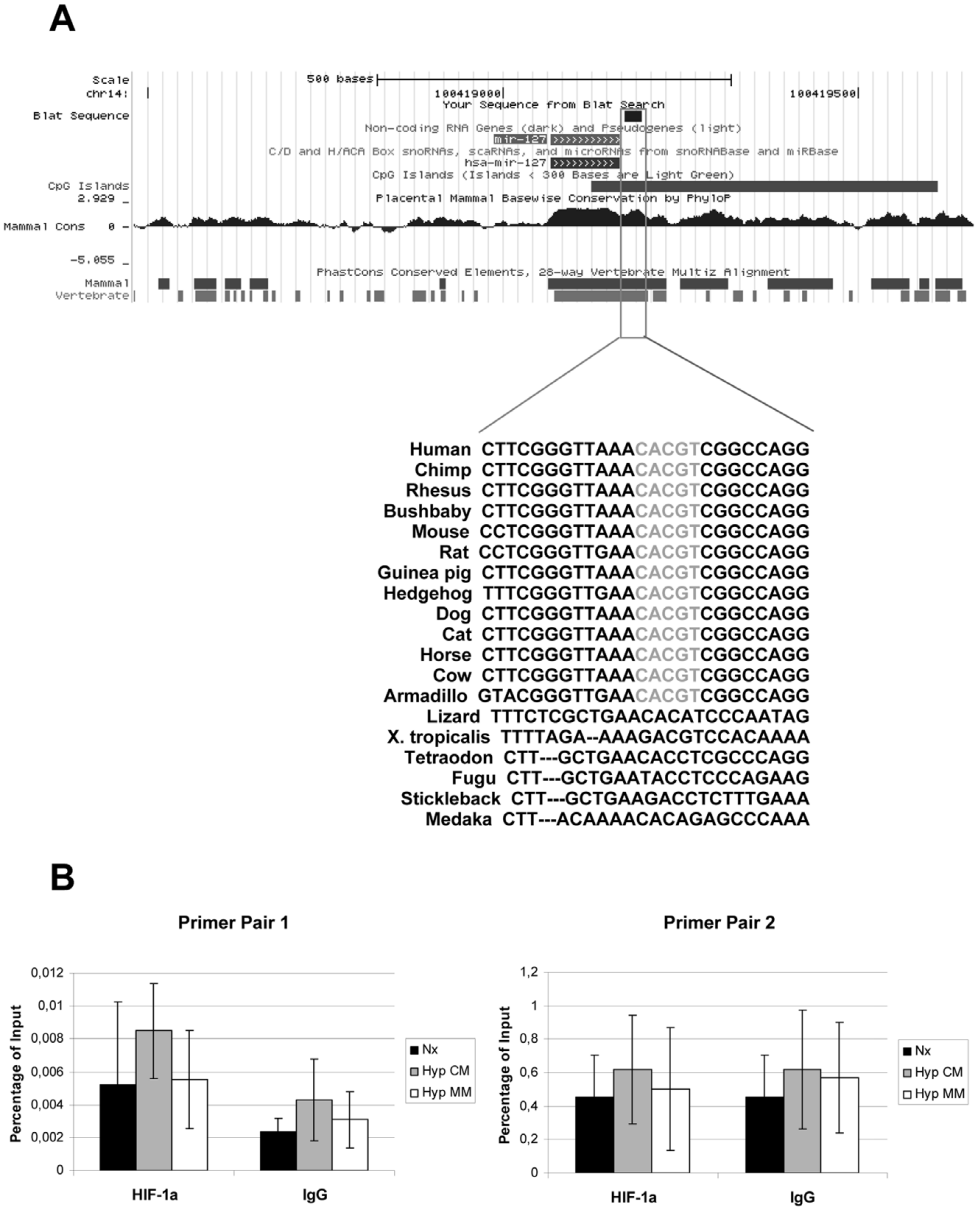
miR-127 gene DNA region presents a putative Hypoxia Response Element. (A) Bioinformatics sequence alignment and conservation studies detected a consensus HRE sequence (CACGT) downstream miR-127 coding region. Alignment map (upper part) shows miR-127 gene and HRE element location into human genomic DNA. Sequence scheme (lower part) indicates that this HRE element is conserved among mammals and several vertebrate species. (B) HRE element inmunoprecipitation was studied by qRT-PCR using two specific primer pairs for the region of interest. Data are presented as mean±SEM of percentage of input of two independent experiments. IgG inmunoprecipitation was used as negative control.

Bioinformatics approaches identified a Hypoxia Response Element (HRE) downstream miR-127 sequence (Figure 3A). This element is conserved among vertebrates and is located in a CpG island, making it a good candidate for miR-127 regulation. However, Chromatin Immunoprecipitation (ChIP) assays demonstrated that HIF-1α does not directly bind to this element (Figure 3B).

Taken together, these data suggest that HIF-1α is a regulator of miR-127-3p in HK-2 cells during H/R, although HIF-1α binding site could not be successfully identified in this study.

### rno-miR-127 Modulation Leads to Changes in Cell Adhesion and Cytoskeleton Structure

**Figure 4:**
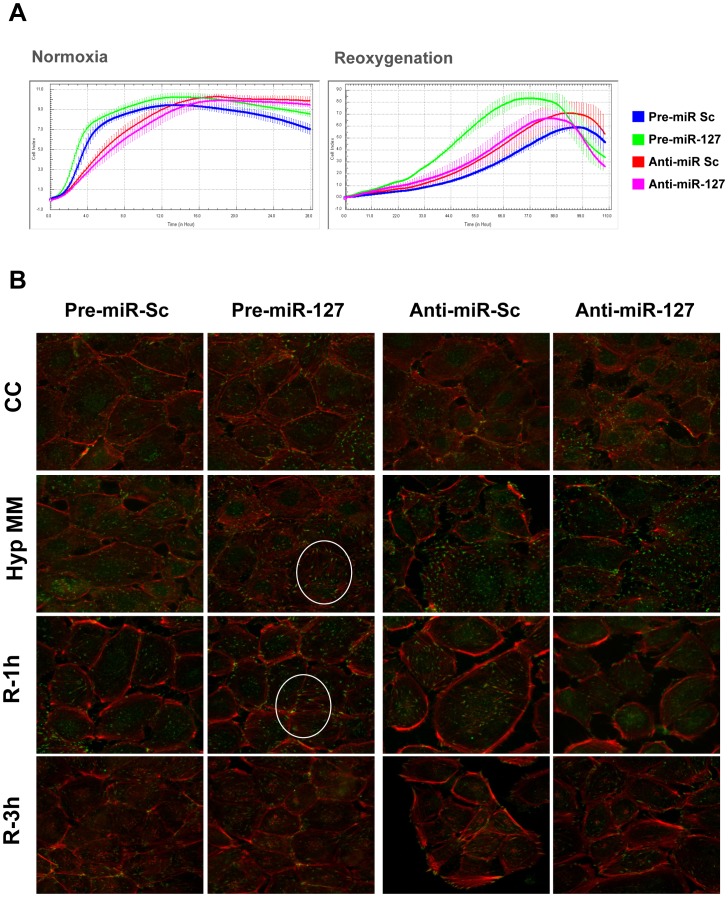
miR-127 overexpression promotes cell adhesion, protects actin cytoskeleton organization and focal adhesion complexes assembly during H/R. (A) NRK-52E cells were transfected with pre-miR-127, anti-miR-127 and their respective scramble control. For normoxic measurement (Left panel), cells were seeded in RTCA plates 24 hours after transfection and real time measurement of cell culture impedance was performed. For hypoxia condition (Right panel) transfected NRK-52E cells were submitted to serum starvation and hypoxia. Immediately after hypoxic treatment, they were detached and seeded in RTCA plates to allow impedance measurement during reperfusion. Each transfection condition was measured in triplicate and cell index is presented as mean±s.e.m. Representative images from 3 independent experiments are shown. (B) Immunofluorescence staining was performed in transfected NRK-52E cells to detect actin cytoskeleton organization (Red) and paxillin localization (Green). Representative confocal microscopy images from three experiments are shown. Co-localization paxilin/actin, as indication of FAC assembly, is marked by circles. (CC: Medium change control; Hyp MM: hypoxia in minimum medium; R-1h: Hypoxia in minimum medium and 1 Hour reoxygenation; R-3h: Hypoxia in minimum medium and 3 hours of reoxygenation;

Based on our previous observations regarding cell adhesion alterations upon H/R [6] and to study the biological significance of miR-127 induction in our system, we performed adhesion assays under normoxia and reoxygenation conditions. Cell adhesion was estimated as monolayer impedance, measured by RTCA device. rno-miR-127 overexpression by pre-miR transfection in NRK-52E cells promotes cell adhesion not only during normoxia but also after hypoxia (Figure 4A).

Thus, we next studied focal adhesion complexes (FAC) assembly in our system by immunofluorescence. miR-127 overexpression protects actin cytoskeleton from disorganization provoked by hypoxic injury (Figure 4B). Moreover, in these samples, paxillin co-localizes with actin fibers (yellow colour in marked circles) indicating FAC correct assembly. Furthermore, rno-miR-127 blockade by anti-miR aggravates cytoskeleton and adhesion structures disorganization caused by hypoxia.

**Figure 5:**
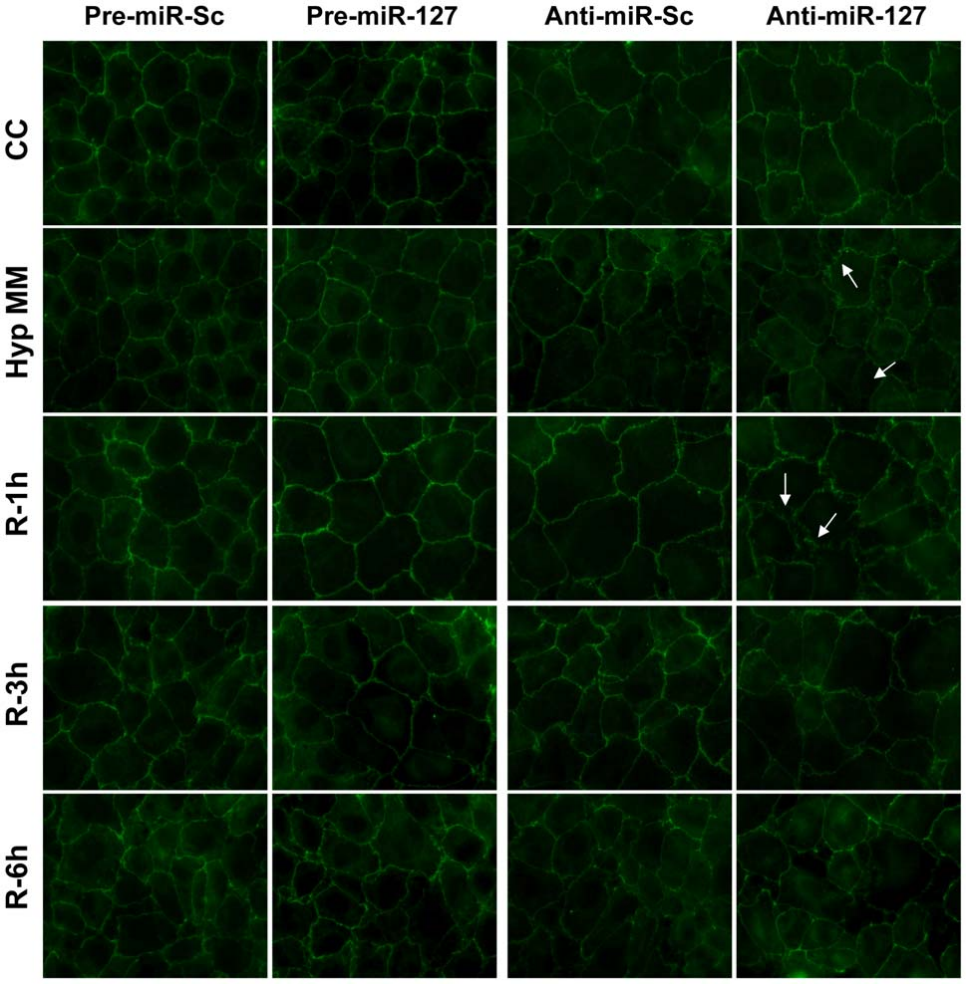
miR-127 overexpression *in vitro* abrogates tight junction disruption during H/R. NRK-52E cells were transfected with pre-miR-127, anti-miR-127 and their respective scramble control. 24 hours after transfection, they underwent H/R protocol. ZO-1 immunofluorescence staining was used to study tight junction integrity. Representative confocal microscopy images are presented from three independent experiments. ZO-1 redistribution from plasma membrane as indication of TJ disruption is marked by arrows. (CC: Medium change control; Hyp MM: hypoxia in minimum medium; R-1h: Hypoxia in minimum medium and 1 hour of reoxygenation; R-3h: Hypoxia in minimum medium and 3 hours of reoxygenation; R-6h: Hypoxia in minimum medium and 6 hours of reoxygenation).

On the other hand, tight junctions (TJ) are essential for epithelial barrier impermeability, thus we investigated rno-miR-127 modulation effects in these structures. Anti-miR transfection clearly enhances hypoxic damage increasing ZO-1 redistribution from the membrane to the cytoplasm, resulting in a discontinuous staining along the membrane and leading to the appearance of gaps among epithelial cells (Figure 5).

All these data demonstrate that rno-miR-127 induction promotes cell adhesion and cytoskeleton structure maintenance during H/R.

### Kinesin Family Member 3B (KIF3B) is a rno-miR-127 Target in Rat Proximal Tubule Cells during H/R

To go further into the biological significance of rno-miR-127 induction, we performed a bioinformatics target prediction for this miRNA using different databases available online, such as microcosm [19] (www.ebi.ac.uk/enright-srv/microcosm), Targetscan 4.1 [20] (www.targetscan.org/vert_40/) and Pictar I [21] (www.pictar.mdc-berlin.de). Only predicted genes present in at least two databases were taken into account. We finally chose KIF3B for further studies because this molecule is involved in cellular trafficking, which is essential for proximal tubule cell function [22] and it is altered in response to H/R.

**Figure 6:**
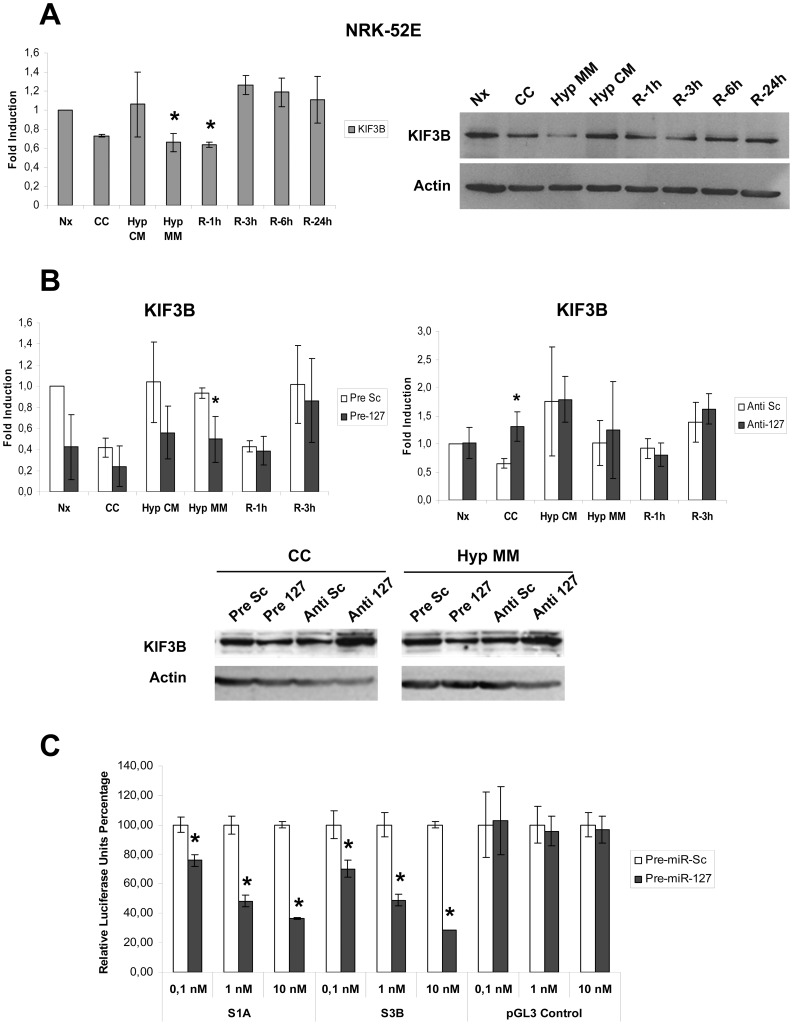
KIF3B is a rno-miR-127 target gene in proximal tubule cells submitted to H/R. (A) KIF3B mRNA and protein levels were estimated in NRK-52E cells submitted to H/R. mRNA levels (Upper graph) were determined by quantitative PCR using 28s mRNA as housekeeping control. Fold changes were calculated using Normoxia (Nx) as basal condition. Data are presented as mean±s.e.m. of five independent experiments and asterisks indicate statistical significance (P<0.05) when each sample is compared to normoxia. KIF3B protein levels were estimated by western blotting using actin as loading control. Representative blot from five different experiments is shown. (B) Study of KIF3B mRNA and protein levels in NRK-52E cells transfected with pre/Anti-miR-127. KIF3B mRNA was estimated by PCR in cells treated with pre-miR-127 (left panel) and anti-miR-127 (Right panel). Fold change were calculated using Nx Scramble as basal condition. KIF3B protein levels were assessed by western blotting using Actin as loading control. Only key points of H/R protocol exhibiting significant changes are presented. Illustrative blots from five independent experiments are shown. (C) Luciferase assays of KIF3B 3′UTR vectors, named as S1A and S3B, and empty vector (pGL3-control). Luciferase activity percentage is represented, using renilla activity as normalization control. Data are presented as mean±s.e.m. of three independent experiments and asterisks indicate statistical significance between Pre-miR-scramble and Pre-miR-127 transfected cells in each condition (P<0.05). (Nx: Normoxia; CC: Medium change control; Hyp CM: hypoxia in complete medium; Hyp MM: hypoxia in minimum medium; R-1h: 1 Hour Reoxygenation; R-3h: 3 Hours Reoxygenation; R-6h: 6 Hours Reoxygenation; R-24h: 24 Hours Reoxygenation).

Firstly we studied the expression of KIF3B in NRK-52E cells during H/R (Figure 6A). KIF3B mRNA is reduced during minimum medium hypoxia and 1 hour of reperfusion, when miR-127 is induced. Similar expression pattern could be observed at protein level.

Moreover, we performed Pre/Anti-miR transfection experiments to determine if modulation of miR-127 could regulate KIF3B expression. Although significant changes were not found for mRNA studies, miR-127 overexpression and inhibition modulate the KIF3B protein (Figure 6B). KIF3B levels are decreased when miR-127 is overexpressed, particularly during Control Condition (CC) and minimum medium hypoxia (Hyp MM).

To further confirm KIF3B modulation by miR-127, KIF3B 3′UTR was cloned into luciferase vectors and mRNA destabilization assays were carried out. Two independent constructions, named as S1A and S3B, were generated to avoid possible side-effects due to mutations undetected by sequencing or other unpredictable effects of cloning procedure (Figure 6C; Figure S3). Data are shown as percentage of luciferase activity reduction by Pre-miR-127 in comparison to scramble (Scramble = 100). miR-127 overexpression significantly reduces luciferase activity in a dose-dependent manner, demonstrating that this miRNA directly regulates KIF3B expression. Therefore, KIF3B is a real target of miR-127 in our system.

**Figure 7:**
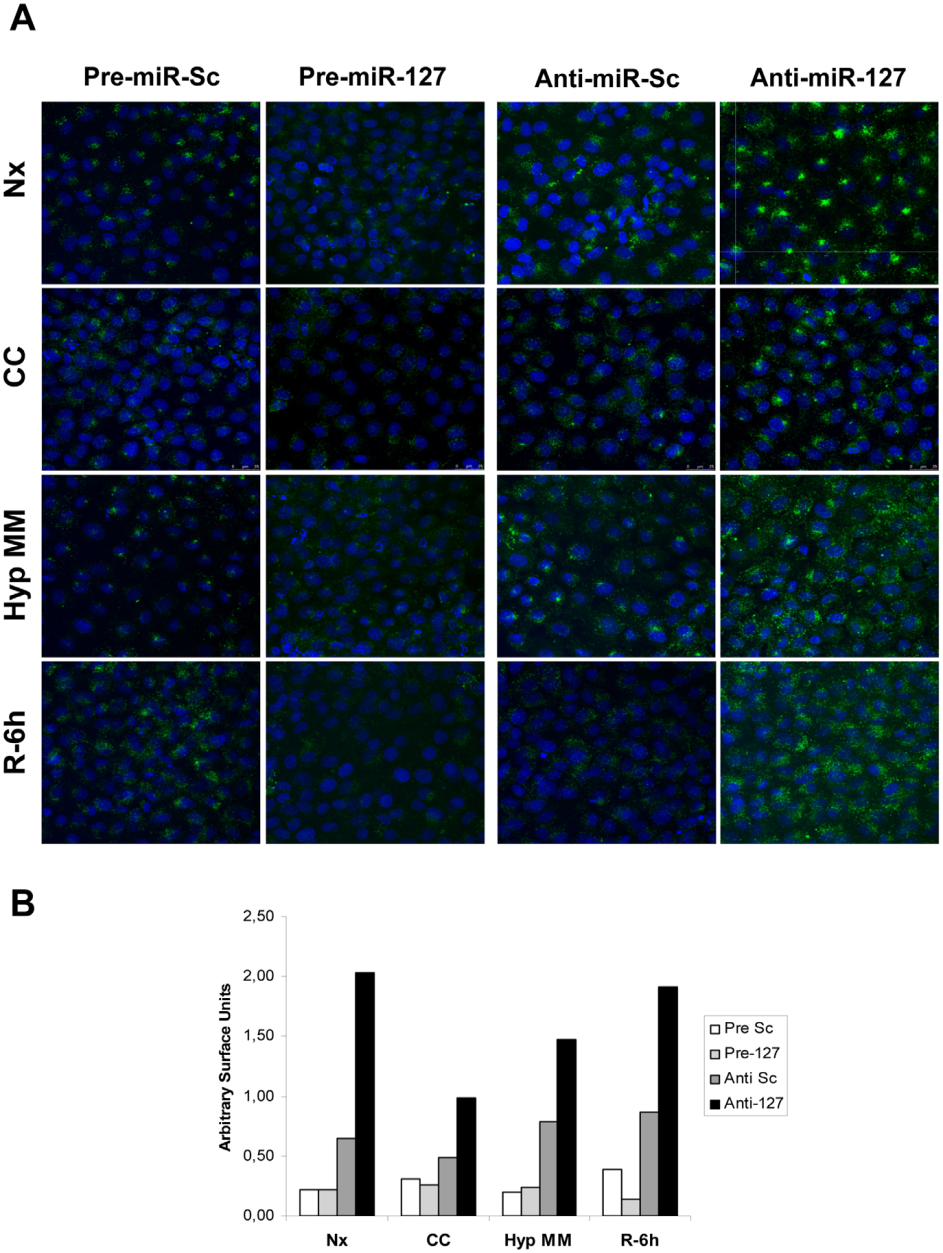
miR-127 regulates trafficking in rat proximal tubule cells in response to H/R. (A)Non receptor-mediated endocytosis assays in NRK-52E cells transfected with pre-miR-127, anti-miR-127 and their respective scramble control. Dextran-FITC of 70 KDa was added to culture medium and nuclei were stained with DAPI. Representative confocal images from five independent experiments are shown. (B) Quantification of previous images expressed as green signal surface/blue signal surface ratio, each signal estimated in pixels^2^. (Nx: Normoxia; CC: Medium change control; Hyp MM: hypoxia in minimum medium; R-6h: Hypoxia in minimum medium and 6 hours of reoxygenation).

Finally, as KIF3B has been involved in endocytosis and microtubular transport in proximal tubule cells [22], we performed non-receptor mediated endocytosis assays in NRK-52E cells transfected with Pre/Anti-miR-127 (Figure 7A). miR-127 overexpression significantly reduced endocytosis activity, whereas miR-127 blockade markedly raises Dextran-FITC internalization. Quantification of Dextran-FITC internalization confirming these results is shown in figure 7B.

These results demonstrate that KIF3B is a target gene for rno-miR-127 in NRK-52E cells during H/R, both regulating proximal tubule cell function.

## Discussion

Identification of molecular mechanisms involved in kidney ischemic injury and recovery is essential for reducing the morbidity and mortality of several habitual clinical practices such as kidney transplant or cardiac surgery. In this work, we have identified rno-miR-127 and its human homologous hsa-miR-127-3p as important mediators of the proximal tubule response to I/R. rno-miR-127 induction during I/R is a cytoskeleton protection mechanism which prevents actin depolimerization and promotes cell adhesion by preventing FAC disassembly and TJ disorganization. Moreover, we have identified KIF3B, a component of kinesin II complex [23], as a real target of rno-miR-127 in proximal tubule cells, with potential implications in cell trafficking.

Several studies have identified miRNAs modulated during renal I/R injury [16], [24] but none have pointed miR-127 as a regulated miRNA in this context. Within our knowledge, this is the first study identifying and characterizing miR-127 in kidney response to I/R. Previous publications have described miR-127 as an ubiquitously expressed microRNA which can be detected in several human and rat tissues including kidney and proximal tubule cells [25], [26]. Moreover, this microRNA is also expressed in other human epithelial cells such as breast [27] and lung [28].

Our results demonstrated also for the first time that miR-127 is regulated by ischemia *in vitro* and *in vivo*. Several microRNAs modulated by hypoxia have been identified [29] but miR-127 was not included among them. Therefore, it is important to notice that our models (*in vitro* and *in vivo*) not only include low oxygen levels, but also serum and nutrient deprivation, which can explain miRNAs different profile between hypoxia and ischemia conditions. In this regard, it has been described that miR-127 is involved in the response of pancreatic cells to glucose availability to produce insulin secretion [30]. In any case, our results indicate a great correlation between the expression of rno-miR-127 in both the *in vitro* and *in vivo* model in rat. rno-miR-127 is increased during hypoxia and Ischemia and at 1h and 24 hours of reperfusion respectively, time points where cellular damage or renal tissue damage is maximum in this model [6], [11]. These results indicate that miR-127 could be a potential renal tissue damage biomarker and it could have a potential role in proximal tubule response to I/R as it will be further discussed.

On the other hand, we observed different miR-127 expression pattern in human cells (HK-2), where its expression is increased mainly during complete medium hypoxia and along reperfusion. Although miR-127 is located in a cluster of microRNAs whose structure is conserved among mammals, miR-127 promoter sequence shows very low sequence conservation between rat and human. Due to this, transcription factor binding sites exclusive for each species could contribute to the observed different regulation [31]. Moreover, in human cells, hsa-miR-127-3p is located in a CpG island which could be submitted to a fine tissue specific regulation by methylation. In several human tissues and in some types of cancer, DNA hypermethylation leads to repression of miR-127 expression [32]. Surprisingly, evidence in rat hepatocarcinogenesis models reveals that hypomethylation of DNA, induced by a methyl-deficient diet, decreases miR-127 expression [33], indicating that although epigenetic DNA regulation can explain differences in miRNAs expression between species, miR-127 regulation by methylation is still controversial.

Furthermore, our interference experiments *in vitro* demonstrated for the first time that HIF-1α is one of the regulators of miR-127 expression, among other mechanisms. Moreover, HIF-1α is induced during H/R and I/R as we have previously demonstrated [11]. A putative HRE element downstream miR-127 sequence, highly conserved among species, was predicted bioinformaticaly. However, CHIP analysis did not confirm HRE functionality. This could indicate that other HRE elements not predicted by the bioinformatic analysis could be responsible for miR-127 regulation in our *in vitro* system. Another possibility is that this regulation by HIF-1α could be indirect. In this regard, a potential crosstalk between c-myc and HIF-1α could be suggested in this case since c-myc is a known regulator of miR-127 expression [34]. Additionally, HIF can modulate the expression of histone demethylases involved in chromatin remodeling [35], which is necessary for the expression of some essential genes for the hypoxic response such as EPO [36] or miRNAs [37].

On the other hand, we identified here for the first time KIF3B as a real target of rno-miR-127 in rat proximal tubule cells during H/R. *In vivo*, not statistical significant regulation of KIF3B was found, probably due to a different regulation of this molecule among all the cell types present in the renal cortex. Proximal tubule cells present a developed endocytosis apparatus involved in urine protein absorption and membrane receptors exposition and recycling [38]. KIF3B has been involved in late endosomes and lysosomes localization [39]. However, recent work has demonstrated that KIF3B is also responsible for receptor and ionic transporter localization in polarized epithelial cell membrane. In this regard, it has been demonstrated that KIF3B knockdown impairs cell polarization in intestine epithelial cells [40] and it is important for the correct localization of kidney anion exchanger 1 and Chloride/proton antiporter CLC-5 in proximal tubule cells [41], [22]. In addition, KIF3B has been unveiled as a specific regulator of constitutive albumin and transferring uptake in polarized kidney cells. KIF3B overexpression promotes internalization of membrane vesicles containing cubilin and megalin receptors, decreasing proximal tubule protein reabsorption capacity [22]. In this work, rno-miR-127 blockade leads to KIF3B overexpression and endocytic activity increase. Thus, miR-127 up-regulation observed during I/R could result in KIF3B downregulation and proximal tubule cell trafficking impairment, as observed during renal I/R injury [42]. Moreover, miR-127 induction and trafficking impair trough KI3FB inhibition could lead to tubular cell protection since cell trafficking requires high levels of ATP, compromised during renal I/R.

Regarding a potential protective role of miR-127 in response to I/R, this work describes for the first time the effects of rno-miR-127 modulation in actin cytoskeleton organization and adhesive structures integrity during I/R injury. miR-127 overexpression prevents FAC disassembly and TJ disruption and epithelial barrier impairment, all of them essential for kidney function. The molecular mechanisms responsible for these effects needs to be further investigated, but the regulation of intestinal TJ permeability by miRNAs has been recently described [43]. Other components of cell-cell adhesion structures such as E-cadherin are also regulated by miRNAs, in particular, miR-200 family. Furthermore, E-cadherin function and AJ integrity could be indirectly regulated by miR-127 target KIF3B. This factor directly interacts with plakophilin-4, which is involved in E-cadherin maintenance in the cell surface and its connection to cytosleketon. Moreover, KIF3B is a regulator of Rho-A activity during stress fibers formation [44], that has been previously observed in proximal tubule cell response to I/R [6]. Therefore, rno-miR-127 induction during I/R and H/R could protect cell-matrix and cell-cell adhesions trough KIF3B downregulation, among other mechanisms not yet identified, contributing to cell structure maintenance and epithelial barrier function.

In summary, we identified for the first time a novel role of miR-127 as a critical regulator of cell-cell and cell-matrix adhesion in proximal tubule cells response to I/R. Additionally we unveiled a new regulation of this miR-127 through HIF-1α. Moreover, a novel target gene for this miRNA was also elicited: KIF3B, with important implications in cell endocytosis. As cell adhesion and cell trafficking are essential for proximal tubule epithelial structure and function, miR-127 and KIF3B could be considered as key molecules for renal ischemic damage management.

## Materials and Methods

### Cell Culture and Hypoxia/Reoxygenation Protocol

NRK-52E cells (ATCC, Barcelona, Spain) were cultured in DMEM containing 10% FBS, 2 mM glutamine, 100 U/ml penicillin and 100 µg/ml streptomycin (Invitrogen, Barcelona, Spain). HK-2 cells (ATCC) were cultured in DMEM/F12 containing 10% FBS, 1 g/l insulin, 0.55 g/l transferrin, 0.67 mg/l selenium (Invitrogen).

For H/R protocol, cells were grown until confluence and then they were serum deprived for 24 hours. For MM hypoxia, monolayers were cultured for 6 hours in HBSS (Invitrogen), in a low oxygen atmosphere containing 1% O2, 94% N2, 5% CO_2_ (Air Liquide, Madrid Spain). For reoxygenation, complete medium was added and plates were placed in a regular incubator with 21% O_2_ [6]. In complete medium hypoxia (Hyp CM), cells were serum starved as described above and then submitted to hypoxic atmosphere for 6 hours. Normoxic cells (Nx) were serum deprived but they do not suffer nutrient and oxygen tension changes. Serum-starved cells following 6 h in HBSS correspond to nutrient depletion control condition (CC) (Figure S1A).

### Ischemia/Reperfusion Model in Rat

Experimental procedures were performed according to the European Community laws (EC609), Spanish guidelines (RD 1210/2005) and approved by the Internal Committee for Animal Ethics of Hospital Universitario Ramón y Cajal. Male Sprague Dawley rats (180–200 g) were anesthetized with an inhaled anesthesia mixture of 2% isoflurane (Abbott Laboratories Ltd. Madrid, Spain) and 1 l/min oxygen. Renal I/R injury was induced after laparotomy by a 45 min bilateral clamping of renal pedicles. Sham operated animals underwent the same surgical procedure without clamping. Animals were sacrificed at 0, 24 h, and at 3, 5 and 7 days after reperfusion. Serum creatinine and urea parameters were estimated using the AEROSET automatic system (Abbot Laboratories). Periodic acid-Schiff staining was performed by slide treatment with 0.5% periodic acid followed by Schiff reactive staining for 15 minutes and then nuclei contrast with Harris haematoxylin.

### miRNA Arrays

RNA was labelled with the miRCury LNA array labelling kit (Exiqon, Vedbaek, Denmark). Labelled samples were concentrated in a SpeedVac, mixed with Exiqon Hybridization buffer, filtered and heated at 95°C for 4 min. Hybridization was performed on miRNA miRCury arrays v8 (Exiqon) in a hybridization chamber at 60°C for 16 hours. Four array replicates per comparison were performed. Images were scanned in a DNA microarray scanner (Agilent technologies, Santa Clara, CA, USA) and analysed with agilents feature extraction software v 9.5. Dye swapping analysis was done with Excel and array assist (Agilent) after data normalization.

### HIF-1α siRNA Transfection *in vitro*

HK2 cells at 80% of confluence were transfected with 100 nM HIF-1α siRNA (sc-44225, Santa Cruz Biotechnologies, Santa Cruz, CA, USA) or scramble siRNA (sc-37007, Santa Cruz Biotechnologies), using Lipofectamine 2000 (Invitrogen, Barcelona, Spain) according to manufacturer’s protocol. Transfected cells were submitted to H/R after 24h of transfection (Figure S1B).

### Pre-miR and Anti-miR Transfection *in vitro*

NRK-52E cells at 90% confluence were transfected with 100 nM anti-miR-127 (AM17000, Anti-miR™ miRNA Inhibitor, Life Technologies, Madrid, Spain) y anti-miR-Scramble (Anti-miR™ miRNA Inhibitors-negative Control #1, Life Technologies). Pre-miR-127 (AM17100, Pre-miR™ miRNA Precursor Molecule, Life Technologies) and Pre-miR-Scramble (AM17110 Pre-miR™ miRNA Precursor Molecules–Negative Control #1, Life Technologies) were transfected with a final concentration of 0.1 nM.

### RNA Extraction and Real-Time PCR

Firstly, kidney cortex was dissected containing a high proportion of proximal tubules. Total RNA was isolated with TriPure Isolation Reagent (11667165001, Roche Diagnostics, Madrid, Spain) by mechanical lysis and Phenol/Chloroform extraction. 2 µg of RNA were used to obtain cDNA using Transcriptor First Strand cDNA Synthesis Kit (04897030001, Roche Diagnostics). 1 µl of cDNA was used as template for quantitative PCR reaction with SYBR Green (11066420, SYBR Green I Master, Roche Diagnostics). Ribosomal 28 s gene was used as housekeeping for data normalization. Primers employed were: *28 s*, forward: CAGTACGAATACAGACCG, reverse: GGCAACAACACATCATCAG; *KIF3B rat*, forward: ATCATACAAACGAGCAGCAG, reverse: GTCTCTTTCAGTTCCAAGGTC; *KIF3B Human*, forward: GCCATTGTAGAGGATCACAG, reverse: CAACAAGCAACTTACTCTCCA.

For microRNA quantification taqman microRNA assays (Life Technnologies) were used following manufactureŕs instructions. RNU6B was used as internal control for data normalization in *in vitro* studies, whereas ribosomic RNA 5s was employed for rat kidney tissue.

### Protein Extraction and Western Blot Analysis

Cell cultures were homogenized in lysis buffer (0.25 M Tris pH = 6.8, 6% SDS, 10% glycerol, 20 mM DTT, Bromophenol Blue, protease and phosphatase inhibitors (Sigma-Aldrich, Madrid, Spain). Homogenates were mechanically disrupted by syringing and then centrifugued. Precleared supernatants were resolved by SDS-PAGE and transferred into nitrocellulose (Hybond-ECL Amersham, GE Healthcare, Madrid, Spain). Primary antibodies used: anti-human anti-HIF-1α, 1∶250 (BD Transduction Laboratories, Madrid, Spain); anti KIF3B 1∶500 (SC-50456, Santa Cruz Biotechnologies). Appropiate horseradish peroxidase-conjugated secondary antibodies (Dako, Barcelona, Spain) were used.

### Immunofluorescence

NRK-52E cells were grown on coverslips coated with collagen IV (1 µg/mL. Sigma-Aldrich). To visualize actin cytoskeleton, cells were fixed in 4% paraformaldehyde, permeabilized with 0.5% Triton X-100, blocked in PBS 1% BSA and stained with Phalloidin-Alexa568 1∶40 (Invitrogen), 30 min at room temperature. For paxillin and ZO-1 inmunostaining, cells were processed as described above and incubated with PBS 1% BSA containing primary antibodies anti-paxillin (Millipore, Madrid, Spain) 1∶250 and anti-ZO-1 (Invitrogen) 1∶200, 1 hour at room temperature. Appropriate secondary antibodies anti-mouse Alexa488 or anti-rabbit Alexa488 (Invitrogen) were used 1∶250 for 1 h at room temperature. Images were obtained with Spectral Confocal Microscope TCS SP5 (Leica Microsystems, Barcelona, Spain).

### Pinocytosis Assay

Dextran-FITC of 70 KDa (SD70S, Sigma-Aldrich) to a final concentration of 1 mg/ml was added to culture medium 6 hours before sample collection and incubation was performed at 37 °C. After incubation, samples were fixed in 4% paraformaldehyde and coverslips were mounted using prolong antifade reagent (Invitrogen) with DAPI. Images were obtained with Spectral Confocal Microscope Leica TCS SP5. Quantification of endocytosis was performed using NIS-Elements BR Image Software (Nikon). For each image, surface of DAPI signal and green signal was estimated in pixels^2^ and quantification was expressed as a Green/DAPI surface ratio.

### Identification of HIF Binding Sites

For the identification of HIF binding sites within the mir-127 locus we follow the strategy described elsewhere [45]. Briefly, we first identified mammal or vertebrate PhastCons elements [46] within the region chr14∶100418481– 100419663 containing the mir-127 gene. Adjacent PhastCons elements were fused if more than 50% of the sequence in the resulting fused region was conserved. We refer to these PhastCons elements located in noncoding regions as conserved non coding sequences or CNSs. Then, we identified conserved RCGTG motifs within these CNSs. A motif was considered conserved when it was present at least in four mammals including human and mouse. Sequences lacking conserved RCGTG motifs were discarded as potential HIF-binding sites (HBS). Finally, sequences containing a conserved motif were scored according to a position specific scoring matrix (PSSM). Aligments and PhasCons elements were downloaded from the UCSC genome browser [47] using the February 2009 (hg19) human genome assembly.

### Cromatin Immunoprecipitation Assay

1×10^7^ HK-2 cells were prepared for each immunoprecipitation. After serum deprivation or hypoxia, crosslinking was performed by adding formaldehyde (1% final concentration).

1×10^7^ cells were resuspended in Cell Buffer Mix (10 mM HEPES/KOH, pH 7.9; 85 mM KCl; 1 mM EDTA, pH 8; 1% NP-40) with 1 mM PMSF and Protein inhibitor Mix (Roche Diagnostics). After centrifugation, pellets were lysed using Nuclear Lysis Buffer (50 mM Tris/HCL, pH 7.4; 1% SDS; 0.5% Empigen BB; 10 mM EDTA, pH 8).

Glass beads were added (Agilent Technologies, Cat: 200069) and sonication was performed in 15 cycles of 10 sec. with 15% amplitude. After sonication, DNA fragments size was checked by agarose gel analysis. Input (5% of the final volume of the lysates) was separated for further determinations.

Protein concentration of each sample was estimated using the bradford assay and then adjusted using nuclear lysis buffer. Then, each sample was diluted 1∶1.5 with Dilution Buffer (20 mM Tris/HCl, pH 7.4; 100 mM NaCl; 2 mM EDTA, pH 8; 0.5%Triton X-100). Preclearing was performed using Sepharose CL-4B beads (Sigma-Aldrich) and incubating for 2 hours at 4°C. After pre-clearing, 2 µg of each antibody (anti HIF-1α Novus 100–134; anti IgG Millipore 07-690) were added and samples were incubated over night at 4°C.

Protein G Agarose beads (Roche Diagnostics) were added to each sample and mixes were incubated for 4 hours at 4°C. After precipitation and washing, DNA was obtained by treatment with Elution buffer (0.1M NaHCO_3_; 1% SDS). Crosslink reversal was performed by adding proteinase K (0.5 ug/uL final concentration) and 200 mM NaCl and incubating at 65 °C over night.

1 µl of purified DNA was used as template for qPCR reaction, using the following primers: Primer pair 1, forward: TCGCTGTGATCACTGTCTCC *; Reverse:* CTGCCACACCCATACTCAGA. Primer Pair 2 *Forward*: TCTGCTTCCTTCGGGTTAAA *; Reverse:* CTAGAGAGGCACGGCATGAG.

### Real Time Monolayer Impedance Estimation

Real Time Cell Analyzer device (Roche Diagnostics) was used for these experiments. 30.000 cells were seeded per well in a final volume of 200 µl. Culture impedance was measured periodically every minute during the first 10 hours, then in 5 minutes-intervals during 24 hours and finally every 10 minutes for 72 hours. Monolayer impedance was expressed as Cell Index, calculated by RTCA Software 1.2.

### KIF3B 3′UTR Cloning and Luciferase Assays

*Rattus norvegicus* KIF3B (NM_001106529) 3′UTR was inserted downstream of the luciferase encoding region of the pGL3-control vector (Promega) using the In-Fusion PCR cloning kit (Clontech-Takara). pGL3-Control vector was digested with XbaI and the KIF3B 3′UTR was amplified from cDNA of NRK-52E cells using specially designed primer pairs which generate 15-bp extremes overlapping with pGL3-control. Schemes of the generated constructions can be found in Figure S3.

For luciferase reporter assays, 125,000 NRK-52E cells were seeded in 24 well plates and transfection was performed at 80% confluence. 1 µg of luciferase plasmid and 0.05 ng of renilla luciferase plasmid per well were transfected using 2.5 µl of lipofectamine. Pre-miR-Scramble and Pre-miR-127 were simultaneously transfected with a final concentration of 0.1, 1 and 10 nMolar. Each transfection condition was carried in triplicate. Luciferase and renilla luciferase activity was measured after 48 hours of transfection using Dual-Luciferase Reporter System (Promega) following manufactureŕs instructions. Luciferase activity was normalized using renilla luciferase values for each sample.

### Statistical Analysis

Data are presented as mean±s.e.m. After the Levene test of homogeneity of variance, the Kruskal–Wallis test was used for group comparison. P<0.05 was considered significant. In case of significant differences, intergroup differences were analyzed by post hoc Mann–Whitney U-tests with the Bonferroni correction. Statistical analysis was carried out using Statistical Package for the Social Sciences (SPSS, Madrid, Spain) version 19.0.

## Supporting Information

Figure S1 **Scheme of** ***in vitro*** **H/R and transfection protocols.** (A) Schematic representation of H/R protocol where changes in nutrients and oxygen tension are indicated for each condition, represented in different arrows. DMEM medium is used for NRK-52E cells, whereas DMEM-F12 is employed for HK-2 cell culture. HBSS is a minimum medium with balanced salt concentration without glucose (B) Transfection and H/R protocol diagram. Cells at 90% of confluence are transfected with HIF-1a siRNA, in the case of HK-2 cells, or pre/anti-miR-127 in NRK-52E cells. 24 hours after transfection, cell cultures undergo H/R protocol as described above. (Nx: Normoxia; CC: Medium change control; Hyp CM: hypoxia in complete medium; Hyp MM: hypoxia in minimum medium; R-1 h: 1 Hour Reoxygenation; R-3 h: 3 Hours Reoxygenation; R-6h: 6 Hours Reoxygenation; R-24 h: 24 Hours Reoxygenation).
(TIF)

Figure S2 **Ischemia/Reperfusion in rats produces renal dysfunction and proximal tubules damage.** (A) Renal function studies in rats submitted to I/R protocol. Serum creatinine and urea were measured for renal function studies. Data are presented as mean±s.e.m. of five animals per condition and asterisks indicate statistical significance compared to Sham condition (P<0.05). (B) Representative PAS staining images of renal tissue during I/R protocol (magnification 100X) (I/R-24 h: ischemia and 24 hours of reperfusion; I/R 3D: ischemia and 3 days of reperfusion; I/R 5D: ischemia and 5 days of reperfusion; I/R 7D: ischemia and 7 days of reperfusion).
(TIF)

Figure S3 **Schematic representation of vectors used in reporter assays experiments.** (A) Scheme of luciferase-KIF3B 3′UTR vectors S1A and S3B. Two independent KIF3B-3′UTR vectors, named as S1A and S3B were. (B) Empty vector (PGL3 control) used as control in luciferase reporter experiments. (C) Renilla luciferase vector used for normalization.
(TIF)
