# Supplementary figures and images for: miR-127 Protects Proximal Tubule Cells against Ischemia/Reperfusion: Identification of Kinesin Family Member 3B as miR-127 Target
Source: PLoS One. 2012 Sep 4;7(9):e44305. doi: 10.1371/journal.pone.0044305 (PMC3433485; doi:10.1371/journal.pone.0044305)

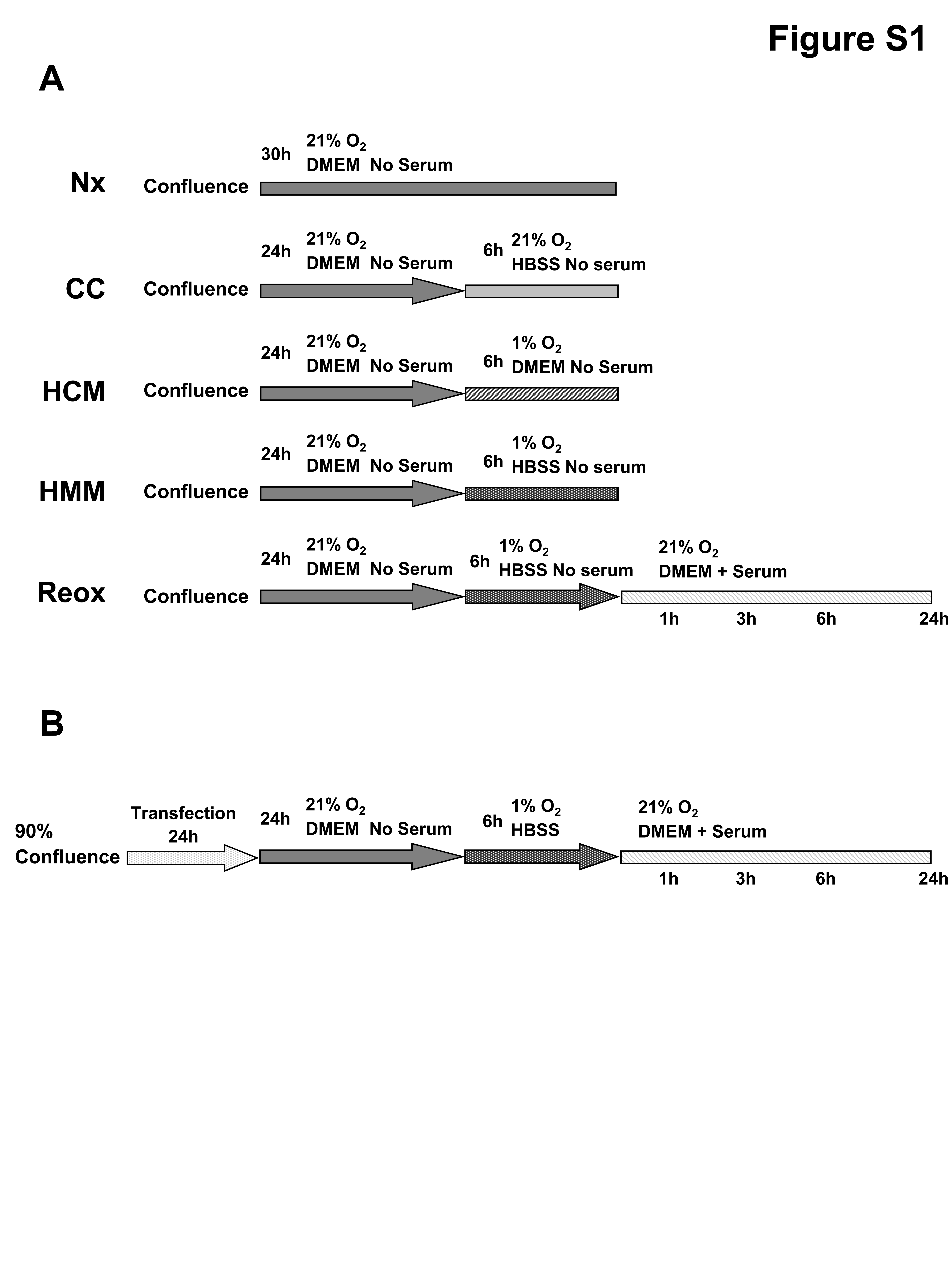

Supplement: Figure S1 — Scheme of in vitro H/R and transfection protocols. (A) Schematic representation of H/R protocol where changes in nutrients and oxygen tension are indicated for each condition, represented in different arrows. DMEM medium is used for NRK-52E cells, whereas DMEM-F12 is employed for HK-2 cell culture. HBSS is a minimum medium with balanced salt concentration without glucose (B) Transfection and H/R protocol diagram. Cells at 90% of confluence are transfected with HIF-1a siRNA, in the case of HK-2 cells, or pre/anti-miR-127 in NRK-52E cells. 24 hours after transfection, cell cultures undergo H/R protocol as described above. (Nx: Normoxia; CC: Medium change control; Hyp CM: hypoxia in complete medium; Hyp MM: hypoxia in minimum medium; R-1 h: 1 Hour Reoxygenation; R-3 h: 3 Hours Reoxygenation; R-6h: 6 Hours Reoxygenation; R-24 h: 24 Hours Reoxygenation). (TIF) [file pone.0044305.s001.tif]

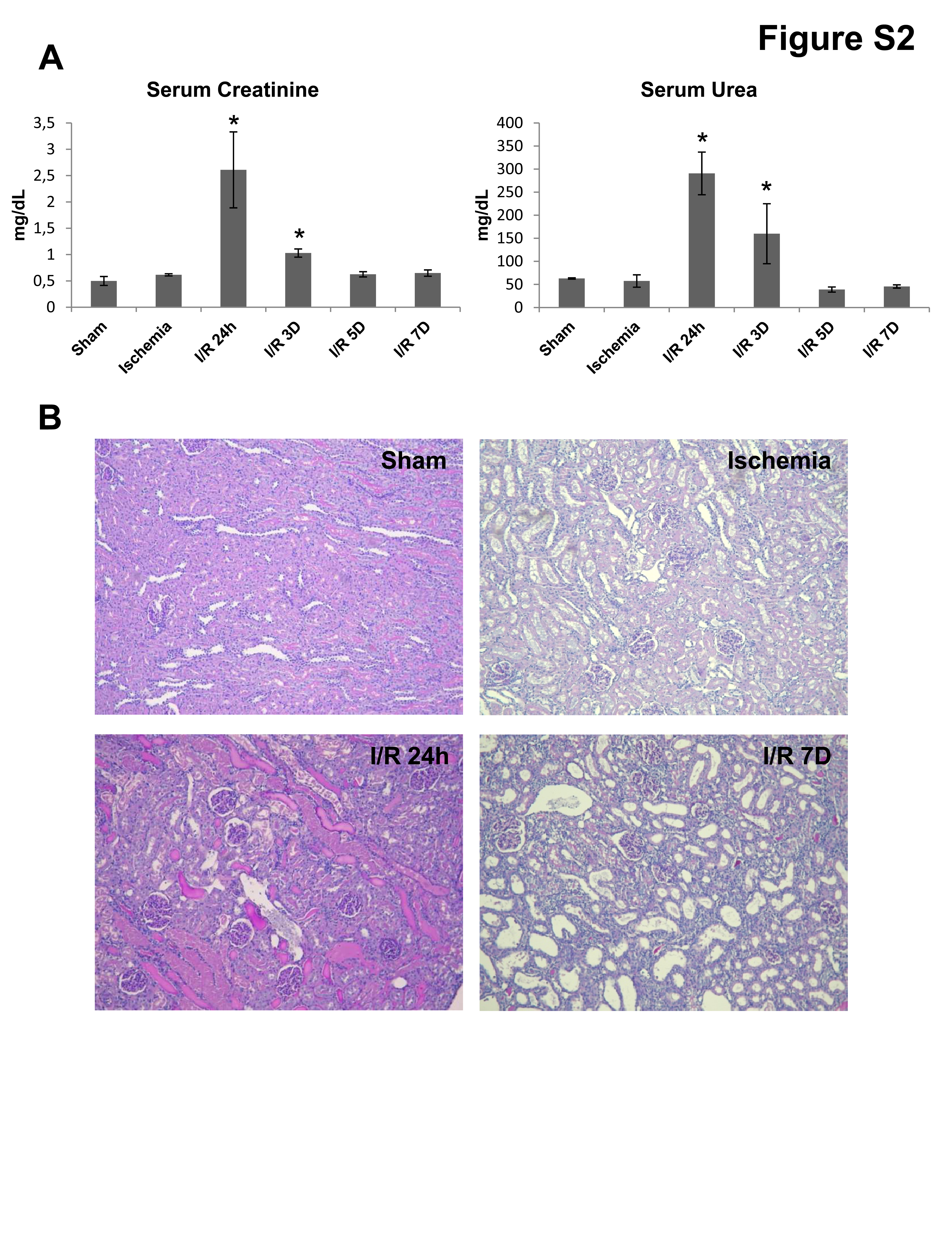

Supplement: Figure S2 — Ischemia/Reperfusion in rats produces renal dysfunction and proximal tubules damage. (A) Renal function studies in rats submitted to I/R protocol. Serum creatinine and urea were measured for renal function studies. Data are presented as mean±s.e.m. of five animals per condition and asterisks indicate statistical significance compared to Sham condition (P<0.05). (B) Representative PAS staining images of renal tissue during I/R protocol (magnification 100X) (I/R-24 h: ischemia and 24 hours of reperfusion; I/R 3D: ischemia and 3 days of reperfusion; I/R 5D: ischemia and 5 days of reperfusion; I/R 7D: ischemia and 7 days of reperfusion). (TIF) [file pone.0044305.s002.tif]

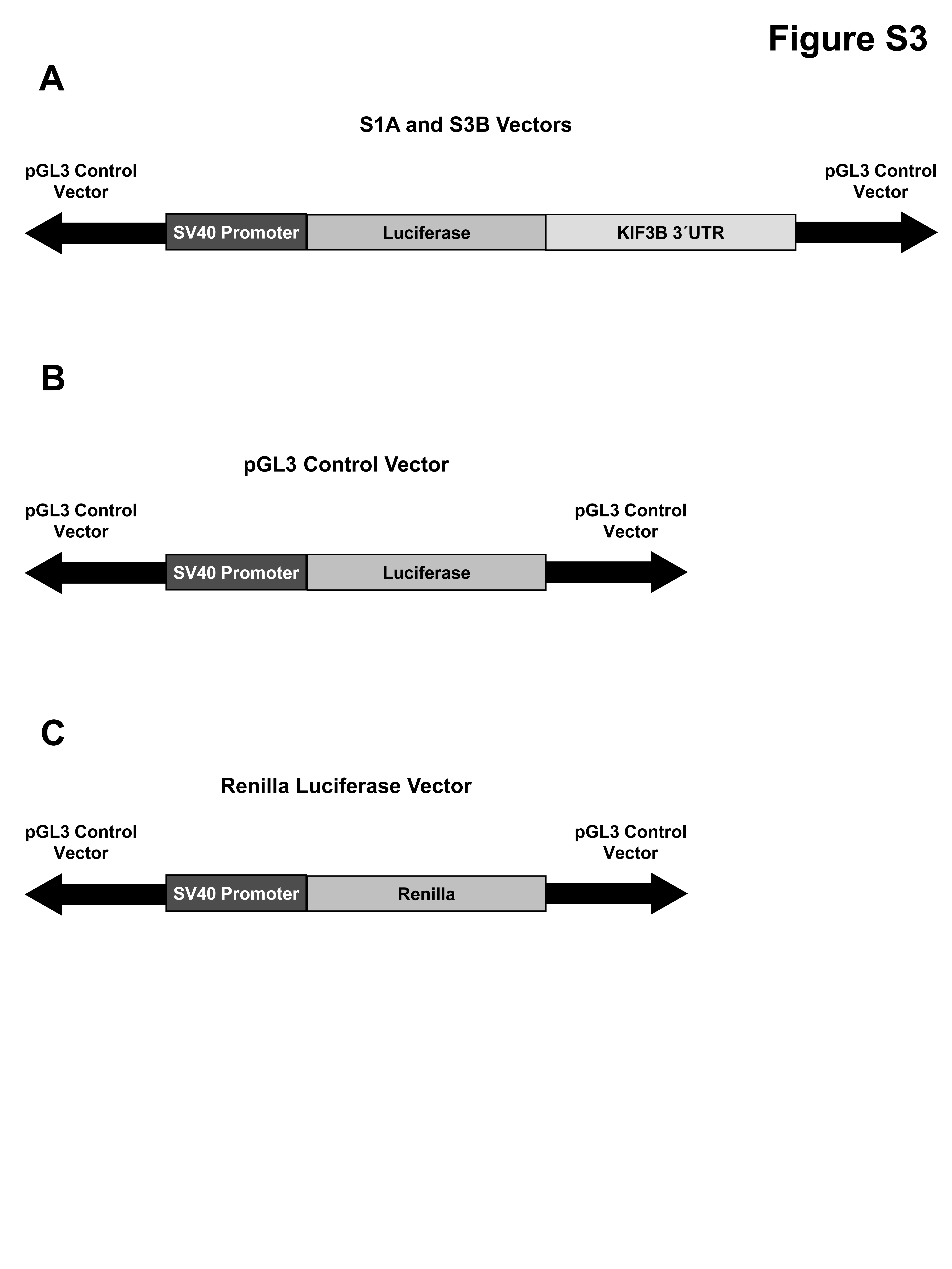

Supplement: Figure S3 — Schematic representation of vectors used in reporter assays experiments. (A) Scheme of luciferase-KIF3B 3′UTR vectors S1A and S3B. Two independent KIF3B-3′UTR vectors, named as S1A and S3B were. (B) Empty vector (PGL3 control) used as control in luciferase reporter experiments. (C) Renilla luciferase vector used for normalization. (TIF) [file pone.0044305.s003.tif]
